# Concomitant illnesses in pregnancy in Indonesia: A health systems analysis at a District level

**DOI:** 10.1371/journal.pone.0279592

**Published:** 2022-12-30

**Authors:** Lareesa M. Ryan, Mohammad Afzal Mahmood, Ismi Mufiddah, Martina Yulianti, Caroline O. Laurence

**Affiliations:** 1 School of Public Health, Faculty of Health and Medical Sciences, University of Adelaide, Adelaide, SA, Australia; 2 Faculty of Medicine, Universitas Airlangga, Surabaya, East Java, Indonesia; 3 Kutai Kartanegara District Department of Health, Tenggarong, Kutai Kartanegara District, East Kalimantan, Indonesia; University of the Witwatersrand Faculty of Health Sciences / Pan African University, SOUTH AFRICA

## Abstract

**Background:**

In LMICs, including Indonesia, there is a rising burden of non-communicable diseases (NCDs) with a prevailing burden of infectious diseases, including among pregnant women. The Indonesian health system faces significant challenges to provide effective care for infectious diseases, and even more so, NCDs. This is concerning due to the greater vulnerability of pregnant women to complications caused by concomitant illnesses (NCDs and infectious diseases), and the need for complex, integrated healthcare between maternal care and other health services.

**Methods:**

The objective of this study was to understand supporting factors and challenges of the health system to providing care for concomitant illnesses in pregnancy and how it may be improved. Semi-structured interviews were conducted with sixteen key stakeholders, including health providers and health service managers, involved in maternal healthcare for concomitant illnesses at a District level in Indonesia. The study was conducted in Kutai Kartanegara District of East Kalimantan. Analysis was conducted using framework analysis to identify themes from transcripts.

**Results:**

Supporting factors of the health system to provide care for concomitant illness in pregnancy included collaboration between health providers and health services, availability of screening and diagnostic tools, and access to universal healthcare coverage and financial subsidies. Common challenges included knowledge and awareness of concomitant illnesses among health providers, competency to diagnose and/or manage concomitant illnesses, and inappropriate referrals. Suggested improvements identified to address these gaps included increasing education and refresher training for healthcare providers and strengthening referrals between primary and hospital care.

**Conclusions:**

The findings identified gaps in the health system to provide care for concomitant illnesses in pregnancy in Indonesia that need to be strengthened. More evidence-based research is needed to guide the implementation of policy and practice interventions for the health system to deal with a broader range of concomitant illnesses in pregnancy, particularly NCDs.

## Background

In the last 30 years, there has been an observed epidemiological transition in the burden of disease for low and middle income countries (LMICs) from infectious diseases, nutritional, maternal and neonatal conditions, to NCDs, as countries develop [1]. However, health systems in LMICs remain fragile and fragmented, and are not well prepared to deal with the changing pattern of disease [2–5]. Some LMICs have made some gains to improve health outcomes through the expansion of Universal Health Coverage (UHC) [6]. However, this has largely concentrated on addressing issues for health services for some infectious diseases, maternal health, and child health [6]. The effectiveness of health systems and health financing coverage is still lagging for NCDs in many LMICs [6]. The World Health Organization (WHO) (2019) estimates that NCDs are responsible for approximately 41 million deaths each year, with 77% of all deaths occurring in LMICs alone [7]. This raises concern as to how health systems in LMICs will provide appropriate and effective health interventions and health services for existing population health issues and the increasing burden of NCDs [6].

Indonesia is an LMIC facing this growing epidemiological shift towards a “double burden” of disease, with high rates of infectious diseases, such as tuberculosis (TB), HIV/AIDs and diarrhoeal diseases, and growing rates of NCDs, particularly ischaemic heart disease, cerebrovascular disease and diabetes [8–10] In Indonesia, 1.365 million (73%) of deaths in the population were attributed to NCDs in 2016 [11]. Significant challenges also exist in the Indonesian health system, such as low quality of primary healthcare, poor integration of care, availability and distribution of the health workforce, inadequate health information systems and lack of access to medical equipment and drugs [12, 13]. Agustina and colleagues (2019) note that the rising “double burden” of disease is putting a strain on Indonesia’s health system to address a broader range of health issues, such as NCDs [12]. Furthermore, it may have implications for Indonesia’s National Health Insurance Scheme, *Jamiman Kesehatan Nasional* (JKN) to achieve complete UHC [10]. Despite Indonesia achieving UHC for over 75% its population in 2018, it still faces many challenges to provide effective coverage of care [12, 14]. In 2019, Indonesia’s health systems performance remains sub-optimal for some particular infectious diseases, such as antiretroviral coverage for Human Immunodeficiency Virus (HIV) and treatment for TB. It is even less effective for treatment of a large number of NCDs, including diabetes, cardiovascular diseases, respiratory illnesses, and cancers. Indonesia’s health system functions as a decentralised system, which contributes to variability in the efficacy of the health system across the country [12]. Significant inequities also exist between districts and provinces, as well as between urban and rural areas, in respect to availability and quality of healthcare services, infrastructure, access to human resources, disease burdens, and health indicators [12].

Beyond the potential implications this may have for the health outcomes in the general population in Indonesia, is the impact that this may have on pregnancy outcomes. Incidence estimates signify a particular growing burden of NCDs in pregnancy, such as diabetes mellitus, circulatory diseases, chronic kidney disease and some cancers [9]. Additionally infectious diseases, such as Human Immunodeficiency Virus/Acquired Immunodeficiency Syndrome (HIV/AIDs) has increased, and rates of TB, sexually transmitted infections (STIs) (excluding HIV/AIDs) and dengue have declined but still remain high [9]. This is particularly concerning as these concomitant illnesses may be aggravated in pregnancy due to physiological changes [15]. For example, diabetes in pregnancy can lead to pregnancy complications such as pre-eclampsia, macrosomia, and intrauterine fetal death [16]. Concomitant illnesses may also increase in severity during pregnancy. For example, the severity of infectious diseases, such as malaria or dengue fever, are significantly higher for pregnant women than compared with non-pregnant women [17]. This is problematic where the health system still struggles with the capacity to provide appropriate care for obstetric-related complications (e.g. haemorrhage, preeclampsia), due to factors such as lack of competency of health providers to diagnose and manage complications, poor quality of care, and ineffective referrals [13]. Concomitant illnesses in pregnancy present another layer of complexity for the Indonesian health system, where they may require different or additional approaches to care such as integrated care with multiple health providers throughout pregnancy, and access to specialist care [18]. However, these services are not always accessible, or health facilities or health workers may not have the competency to address such complex diseases in pregnancy [18–20]. Therefore, there is a need to understand how the health system in Indonesia needs to improve to prepare to deal with the growing “double burden” of disease. This study aims to understand current strengths and challenges of the health system, and how it may be improved by focusing on concomitant illnesses in pregnancy.

## Materials and methods

### Study setting

This study was conducted in the district of Kutai Kartanegara, in the province of East Kalimantan. In 2020, Kutai Kartanegara district had a total population of 696,784 [21]. The district consists of 18 sub-districts (*kecamatan*), with approximately 105,000 people living in the urban capital city of Tengarrong. The rest of the district is predominantly rural, with populations of sub-districts ranging from 8,600 to 66,000 [21]. The district department of health governs three hospitals, with the main tertiary hospital located in Tengarrong, and the other two frontline referral hospitals located rurally approximately two hours away [22]. In respect to primary care, it governs 32 primary health care centres (*puskesmas*) (22 with in-patient services), 17 mobile puskesmas (*pusling or Puskesmas Keliling)*, and 175 sub-health centres (sub-*Puskesmas*, health posts and clinics (see S1 File, village level)). In 2020, there were 12,862 livebirths and a maternal mortality ratio of 233 maternal deaths per 100 000 livebirths [21]. An overview of Indonesia’s health system structure is provided in S1 File.

### Study design

This qualitative study employed a pragmatic approach. Pragmatism provides a methodological approach whereby the methods selected are those most suitable and practical to answer the research question, without being directed by a specific paradigm or worldview [23].

### Participant recruitment

Key informants were recruited from a cross-section of groups involved in the management and delivery of maternal healthcare for concomitant illnesses in the district. Participants were identified by local research collaborators and invited to participate in the study. Inclusion criteria for participants included a minimum of 5 years’ experience in their type of position in maternal health, and a minimum of 3 years’ experience working in Kutai Kartanegara district. A list of potential healthcare providers identified were representative of primary care facilities/hospitals from different urban and rural sub-districts within the district. Potential health service managers identified were representative of varying management roles fundamental to delivering care for concomitant illnesses in pregnancy across the whole district. An invitation email, including participant information forms describing the purpose of the study, were sent to potential participants. Participants were provided the opportunity to ask questions about the study prior to providing consent.

### Data collection

Semi-structured interviews were conducted utilising an interview guide, tailored according to participant groups (health providers or health service managers). An overview of the interview guide is provided in the S2 File. The guide was translated, piloted with a health provider, and amended to ensure questions conveyed there intended meaning. As part of the introduction of interviews, it was explained to respondents that the focus of the study was on health system level practices, not practices or clinical skills of individual health providers or health services. Of the interviews, LR conducted interviews directly in English (n = 3) or with an interpreter in Bahasa Indonesian (n = 13). Duration of interviews lasted approximately 1 to 2.5 hours. Interviews were conducted using videoconferencing software, with participants and interpreters situated in private places. Written consent and permission for digital recording were sought from participants prior to commencement of interviews. Participant recruitment and data collection were conducted from 15^th^ August 2020 - 5^th^ March 2021. Participant recruitment and data collection was ceased based on data saturation according to participant groups or unavailability of the participants.

### Data analysis

Interviews conducted in English were transcribed by LR. Interviews conducted in Bahasa Indonesian were transcribed using a transcription service. Transcripts were analysed applying the Framework Analysis Method [24], using NVivo 12 Plus. The working analytical framework utilised the priori themes of the strengths, challenges and suggested improvements for the health system to organise and analyse the identified themes and sub-themes. Samples of two transcripts were shared with two research team members, CL and MAM, for review of initial coding. The working analytical framework was shared with two research team members, CL and MAM, for review and refinement of coding in reflection of the themes and sub-themes identified. Preliminary findings from the data analysis were shared with research team members in Indonesia for their validation of the themes and sub-themes identified. This study has been reported guided by the Consolidated Criteria for Reporting Qualitative Research (COREQ) criteria [25], as provided in S3 File. An author positionality statement is also reported at S3 File.

### Ethics

Ethical approval was granted for this study by the Human Research Ethics Committee of The University of Adelaide (H-2020-112) on the 9^th^ July 2020.

## Results

### Participant characteristics

Table 1 provides an overview of the characteristics of the 16 participants involved in this study.

**Table 1:** Characteristics of participants.

Job function	Participants	Mean years of experience in role (SD; Range)	Administrative level	Location (Urban/Rural)
Health service managers	3	11.3 (0.57; 11–12)	District (n = 3)	Urban (n = 3)
Obstetricians	3	9.5 (4.58; 7–12)	District (n = 1) Sub-district (n = 2)	Urban (n = 2) Rural (n = 1)
Specialists	2	8.5 (3.53; 6–11)	District (n = 2)*	Urban (n = 2)
Head of Primary Health Care Centres (Puskesmas)	2	17.5 (2.12; 16–19)	Sub-district	Urban (n = 1) Rural (n = 1)
Midwives	3	20.0 (54.35; 15–23)	Sub-district	Urban (n = 1) Rural (n = 2)
Midwife coordinators	4	12.5 (5.44; 5–18)	Sub-district	Urban (n = 2) Rural (n = 2)

### Themes and sub-themes

The themes and sub-themes identified are presented around the study’s aim to understand the preparedness of the health system to provide care for concomitant illnesses in pregnancy, including its strengths and challenges, and areas for improvement. This included three themes and nineteen sub-themes (see Table 2). Some findings identified were specific to the health system with reference to concomitant illnesses in pregnancy, whilst others are related to the broader context of the health system.

**Table 2:** Themes and sub-themes generated from the interviews.

Themes	Sub-themes
Challenges	• Knowledge and awareness of concomitant illnesses among health providers • Competency to diagnosis and/or manage concomitant illnesses among health providers • Lack of appropriate referrals from tertiary to primary care • Self-care and healthcare seeking behaviours of pregnant women with and without concomitant illnesses • Lifestyle and dietary behaviours of pregnant and reproductive-aged women • Capacity of primary healthcare services
Supporting factors	• Collaboration between health providers and health services for concomitant illnesses in pregnancy • Availability of screening and diagnostic measures for concomitant illnesses in pregnancy • Access to universal health coverage and financial subsidies
Suggested improvements	• Education and training for health providers • Strengthening integration of care • Increasing health education • Strengthening family planning • Development of protocols and guidelines • Improving capacity and role of primary healthcare facilities • Increasing human resources • Improving access to physical resources • Strengthening management and leadership • Increasing health financing

### Challenges

A key health system challenge identified by participants was the lack of competency of the healthcare workforce to provide care for concomitant illnesses in pregnancy. This included knowledge and awareness, competency to diagnosis and/or manage concomitant illnesses, and a lack of appropriate referrals.

#### Knowledge and awareness of concomitant illnesses

A majority of stakeholders held the perspective that health providers had a greater awareness and knowledge of obstetric-related complications, such as haemorrhage, hypertension or preeclampsia than those related to concomitant illnesses. This was attributed to obstetric-related complications often being sudden, more clearly identifiable to diagnose, or its requirement for an emergency response due to higher risk of fatality, compared to concomitant illnesses. As a midwife coordinator stated:

*Health providers are more heedful of direct causes like hypertension and haemorrhage–and the handling is therefore more efficient. I’d say treatment of concomitant illnesses isn’t as comprehensive and might be considered less important than maternal health cases such as hypertension (Midwife coordinator 1)*.

#### Competency to diagnose and/or manage concomitant illnesses

Some participants reported that concomitant illnesses may not be detected or are diagnosed late in pregnancy. An obstetrician commented that there are many women being diagnosed with concomitant illnesses for the first time when they arrive at hospital for delivery or seeking care for another issue:

*For those that aren’t aware [they have a concomitant illness], it’s difficult, because the patient only finds out at the ANC [antenatal care] check-up [at hospital], when they might have a complaint or existing cough… Or for those with diabetes mellitus, with high blood sugar, we will find it when the mother is ready to deliver at hospital [for surgery], and they will be immediately referred to the internist (Obstetrician 1)*.

In addition, in some instances it was noted women’s concomitant illnesses were not detected, therefore were managed too late in pregnancy, leading to maternal or foetal complications. A midwife recounted an example of late detection: *This year we had a patient with TB pass away*. *The TB wasn’t detected during pregnancy*, *and it was only after premature labour at twenty-eight weeks*, *that we noticed an increasing cough and eventually the existence of TB (Midwife 1)*.

Some midwives and health service managers also noted that some providers involved in ANC and delivery lack the necessary competency and training to manage concomitant illnesses as well as obstetric complications. Part of this related to the type of health provider and to the location of services. The involvement of traditional (non-formally trained) birth attendants (*dukuns*) for deliveries, who are not competent to manage concomitant illnesses due to lack of training, was seen as an ongoing challenge. This was expressed from the perspective of a midwife: *There are some dukun that deal with concomitant illnesses*, *heart problems and pre-eclampsia*. *The dukun assists with that*, *which can lead to the mismanagement of some patients (Midwife 1)*.

Health service managers also described the variability in competency of midwives to manage concomitant illnesses and obstetric complications in pregnancy. One reasons for the difference in competency was attributed to the lack of training opportunities in rural areas:

*There is a record that midwives move to urban areas to increase their knowledge by training. They have some opportunities to increase their knowledge but for midwives in rural areas they have little opportunities, so I think that is the variation that could happen (Health Service Manager 3)*.

#### Lack of appropriate referrals

Several midwife coordinators and midwives based in primary care described the challenge to provide follow-up care to pregnant women with concomitant illnesses. This was due to poor facilitation of hospital to primary care referrals after hospital care/visits. It was also noted that when there was a referral back to primary care from the hospital level, women often were provided with a lack of or incomplete documentation of the hospital care provided, particularly related to their concomitant illness. A midwife reflected on this challenge: *There is [an] issue of return referrals sometimes coming back to the clinic without any details being recorded in the referral letter–details of medication*, *dosage and future referral details (Midwife 3)*. Primary care providers noted that information on the treatment provided at a hospital level for the management of their concomitant illnesses was often not recorded in women’s Pink Books (personally-held maternity records in Indonesia), which impeded the provision of appropriate care for pregnant women returning for ANC.

#### Self-care and healthcare seeking behaviours of pregnant women with and without concomitant illnesses

In respect to self-care and health seeking behaviours of pregnant women, participants raised the challenge of patient compliance with medical advice. Most participants across all groups raised the concern of pregnant women with concomitant illnesses not seeking care in hospitals for their delivery, despite medical advice. Some respondents highlighted that this was also a challenge across pregnant women generally in the district. A midwife coordinator recounted the story of a mother with a concomitant illness not adhering to medical advice to deliver in hospital:

*Two years ago, there was a patient with diabetes mellitus who had already entered labour, despite having previously agreed to deliver at the hospital. We referred her to the hospital regardless, and the delivery was somewhat fraught considering how the baby was overweight, around 4.3 kilograms… The patient was scared. From early on we had directed her to give birth at the hospital because of high blood sugar and the possibility of the baby being overweight. But she still came to us at the clinic, not wanting to be referred, because she feared the prospect of surgery. Finances weren’t a problem–she was covered by the insurance policy. She was just scared about undergoing an operation (Midwife Coordinator 3)*.

In addition, some participants highlighted that compliance with medical advice extended to patient’s not adhering to medication schedules, particularly where pertinent to control and manage their concomitant illnesses, such as TB or diabetes mellitus. A specialist highlighted this issue with reference to insulin use: *Sometimes we have the difficulties from the [pregnant] mothers or from the locals*, *[which] they do not want to use the insulin*, *they just don’t want to inject themselves*, *maybe*. *They [only want to manage] the diabetes mellitus [with] oral medication*, *however [by the protocol] we must use insulin*, *[requiring] injection [by the patient] on their own in the home (Specialist 1)*. Participants suggested that socio-economic and cultural factors may partly explain why it may be difficult for women to adhere to medication or access care. Socio-economic and geographical factors included low education levels, a lack of access transport and living in remote locations. Cultural stigma associated with certain concomitant illnesses, such as TB and HIV, is also a barrier for some pregnant women accessing care in the district, as explained from the perspective of an obstetrician:

*The community here still believes that [people] with HIV will… become isolated… shunned by the community. So, that makes it harder for us when it comes to further treatment because they’ll certainly cut themselves off. (Obstetrician 3)*.

#### Lifestyle and dietary behaviours of pregnant and reproductive-aged women

The contribution of NCDs to the burden of concomitant illnesses in pregnant and reproductive-aged women was also highlighted as a growing challenge in Indonesia. Participants linked this rise in NCDs in this group of women to lifestyle and dietary behaviours. Some participants attributed this to existing cultural eating habits of salted fish from the rivers in the district of Kutai Kartanegara, whilst others attributed it to the rise in consumption of processed foods and drinks with high carbohydrate and sugar contents, increased accessibility to such foods due to home delivery, and a lack of exercise. One Head of Puskesmas commented on this change in dietary behaviour of pregnant women:

*These days, you can order anything straight to your door, which is changing the way pregnant women consume food. If you buy something, we won’t necessarily know how much sugar it contains, and whether it’s natural or artificial (Head of Puskesmas 1)*.

#### Capacity of primary healthcare services

The capacity of primary healthcare to provide health services for infectious diseases and NCDs for the general population and pregnant women was identified as sub-optimal, particularly in sub-health centres (sub-*Puskesmas*, health posts and clinics (see S1 File, village level)). A health service manager pointed out the comparison of the quality of primary health care services available in sub-health centres to that of Puskesmas:

*It’s difficult sometimes to establish a strong founding at the sub-health centre, because sub-health centres are often overlooked by programs; many midwives aren’t given the chance to attend seminars and training. In terms of quality of service… at the sub-health centre ANC isn’t properly maintained, in contrast with higher quality of care at Puskesmas (Health service manager 2)*.

In sub-health centres, some participants attributed this to a lack of workforce, resulting in delayed screening or lab results and ineffective integration between multiple health programs. The other issues raised by participants included a lack of access to infrastructure in sub-health centres and rural/remote villages, including screening equipment and a lack of supply of medications. A Head of Puskesmas commended on the implications that a lack of access to screening equipment in sub-health centres has for pregnant women with concomitant illnesses:

*When we encounter a pregnant woman with diabetes mellitus, and when checking blood sugar levels and so on, one limitation is that equipment for blood sugar inspections, for example, might not have penetrated to the assisting clinic level–it might only be at the larger clinics [puskesmas]. … In some villages, the village itself assists in raising money to fund inspections (Head of Puskesmas 2)*.

In addition, some health providers also highlighted the barriers to reaching patients in remote areas to provide primary health care, with many pregnant women only accessible by river.

### Supporting factors

Despite challenges of the health system identified by participants, there were also some supporting factors for managing concomitant illnesses in pregnancy, including collaboration between health services and health providers, and the availability of screening and diagnostic tools. In a broader context, access to health financing was a key supporting factor for providing care for pregnant women.

#### Collaboration between health providers and health services for concomitant illnesses in pregnancy

Various participants’ reported the collaboration between primary and hospital settings and within levels of care or health facilities as a supporting factor for the management of concomitant illnesses in pregnancy. Obstetricians and specialists reported on the collaboration between them and primary care providers in managing pregnant women with suspected or pre-existing concomitant illnesses. This included primary care providers consulting with them via telemedicine or social media (WhatsApp) regarding results from screening tests and examinations and for advice on patient referrals. Also, obstetricians noted that collaboration with other specialist colleagues at a hospital level supported case management due to availability of staff, co-location and ease of the referral process. Primary healthcare providers reported that collaboration supports them in delivering care between health programs within primary care; enabling village community health workers (*kader*) to connect high-risk pregnant women with health service and programs and to monitor their compliance with medication schedule; and for facilitating referrals to hospital providers. The strength of program integration within the level of primary care is illustrated in the following quote: *Whether it’s a case of diabetes mellitus*, *TB*, *HBsAg (Hepatitis B) or HIV*, *it’s handled effectively owing to the program integration… If that’s conducted properly*, *it’s very effective*. *The system is ready for anything that crops up*, *the only variable is the adeptness of staff (Head of Puskesmas 2)*.

#### Availability of screening and diagnostic tools for concomitant illnesses in pregnancy

Availability of screening and diagnostic tools in primary care for concomitant illnesses in pregnancy was identified as a supporting factor of the health system to identify these patients. Several primary healthcare providers across participant groups and some specialists emphasised the importance access to screening for concomitant illnesses (in primary care and by field visits from specialists) plays in detecting HIV, hepatitis, diabetes mellitus, syphilis, TB and hypertension (for heart disease) in pregnancy. From the perspective of a couple of midwife coordinators, they noted that the increased availability of screening tools in health facilities in recent years in Indonesia has led to an increase in detection of some conditions, such as hepatitis, syphilis, HIV and diabetes. This was illustrated by the following quote: *It’s possible that*, *previously*, *because facilities used to be insufficient*, *screenings for diabetes and the like didn’t pick up as many cases–so*, *recorded cases are going up*, *yes (Midwife coordinator 1)*.

#### Access to universal healthcare coverage and financial subsidies

More broadly, a key supporting factor of the health system was access to health financing for pregnant women with and without concomitant illnesses. Several participants described the contribution of Indonesia’s national health insurance scheme (JKN) administered by *Badan Peyelenggara Jaminan Sosial* (BPJS) to improving access to primary care provided by dedicated health programs, such as for illnesses like TB, or referrals for hospital care. This included expenses being covered by UHC for consultations for diagnosis, x-rays, ultrasounds, medications, and in-patient hospital care for women both with and without concomitant illnesses, particularly for women from low socio-economic status groups compared to previously: *We can perform any lab that we have*, *we don’t limit [that] from the hospital*. *So*, *I don’t have to think*, *‘oh this patient can’t afford the bills for the examination’*. *No*, *I have never thought about that… because the facilities are available for every patient (Obstetrician 2)*. Financial subsidies from community village funds or *JAMPERSAL* (community health insurance for ANC, childbirth and postpartum care) were also highlighted as co-supporting mechanisms to assist women from low socioeconomic groups to access care, nutritional support, and medications, in conjunction with BPJS: *If a particular patient’s family is poor*, *we are assisted by the Village Funds Allocation [Alokasi Dana Desa*, *or ADD] and coordinate well with villages (Midwife 3)*.

### Suggested improvements

Participants offered a range of suggestions as to how the health system may improve to provide care for concomitant illnesses in pregnancy (see Table 3). These included education and training for health providers, strengthening integration of care, increasing health education, strengthening family planning, development of protocols and guidelines, improving capacity and role of primary healthcare facilities, increasing human resources, improving access to physical resources, strengthening management and leadership, and increasing health financing. These suggestions reflected improvements at a district level in Kutai Kartanegara, as well as at a provincial or national level in Indonesia.

**Table 3:** Suggested improvements to the health system to provide care to concomitant illnesses in pregnancy and to address the issue in a broader context.

Suggested improvements	
Education and training for health providers	• Specialised training workshops and more routine refresher courses for concomitant illnesses, particularly for midwives • Increasing training in midwifery academies for concomitant illnesses • Improving communication skills of health providers • Standardised medical approaches for all providers • Inclusion of midwives at sub-clinics in training • More rigorous supervision in primary care
Strengthening integration of care	• Strengthening referrals between primary care and hospitals • Specialised and standardised referral protocols specific to concomitant illnesses • Strengthening integration between maternal healthcare providers and health programmes with support from government and Ministry of Health • Increasing utilisation of telemedicine • Strengthening partnerships between primary care and village level stakeholders, including community health workers (kader) traditional birth attendants (dukun) and village heads
Increasing health education	• Expanding health promotion to NCDs in health programmes, particularly for pregnant women • Increasing health education to address fears of mothers and families from obtaining treatment or delivery at hospitals
Strengthening family planning	• Strengthening pre-pregnancy and pre-marital counselling to delay or prevent pregnancy for women with concomitant illnesses • Strengthening provision of long-acting reversible contraception or sterilisation • Strengthening health promotion of family planning methods, including after birth
Development of protocols and guidelines	• Standardisation of protocols and guidelines across primary health services • Development of practice guidelines and referral protocols specific to concomitant illnesses • Expanding integrated guidelines used for COVID-19 pregnancies in hospitals to other health conditions as a model • Guidelines for health insurance for high-risk cases • Policies safeguarding dukuns
Improving capacity and role of primary healthcare facilities	• Strengthening screening for concomitant illnesses • Increasing frequency of ANC visits above standard visit model • Improving initiation of first ANC visits • Provision of medication (i.e insulin) usually dispensed at hospitals to minimise costs and transportation barriers for women • Strengthening delivery of specialist consultations and advice by telemedicine
Increasing human resources	• More lab analysts to strengthen screening and reduce wait times in primary care • Scheduled screening visits at sub-clinics with specialists • Appointing midwives in villages without healthcare staff • Improving timely access to specialists in hospitals • Increased health workforce reflective of disease trends
Improving access to physical resources	• Increasing availability of equipment in regional areas, including ultrasound equipment, screening tools, ambulances • Increasing medication stocks in regional areas
Strengthening management and leadership	• Improving monitoring and evaluation of programme integration and services, particularly with support from Ministry of Health • Strengthened engagement at Ministry level including Ministry of Education and Culture and Ministry of Religion to address family planning and NCDs, particularly for adolescents • Better coordination between health departments from national, provincial, to district level
Increasing health financing	• Uncapped or increased health financing of health insurance for high-risk pregnancies with concomitant illnesses • Increased funding to facilitate comprehensive care required for infectious diseases and NCDs more broadly

## Discussion

This study has identified challenges and strengths of the health system at a district level to provide care for concomitant illnesses in pregnancy, and potential strategies to how it can address the growing “double” burden of disease in pregnancy. The key findings and suggested improvements to the health system are discussed below in the context of the existing evidence base, with reference to the WHO Health System Building Blocks [26].

From the perspective of service delivery, our study participants recognised that collaboration between health providers within level of cares or health facilities, and between primary-hospital health providers are factors that support providing care for pregnant women with concomitant illnesses. However, integration between primary care and hospital settings remains sub-optimal for the screening, diagnosis, referral and management of pregnant women with concomitant illnesses. Inappropriate referrals and inadequate integration of care are known contributing factors to poor pregnancy outcomes in Indonesia, including for concomitant illnesses [18, 19, 22]. Integration of care between maternal healthcare and other health services is an important factor that needs to be addressed given the complexity of the management of concomitant illnesses in pregnancy, and the magnitude of health providers involved in service delivery along the continuum of care from pre-conception, ANC, intrapartum care and postnatal care in Indonesia [18]. One suggested solution to strengthen integration of care by participants included development of specialised referral protocols. In respect to specialised referral protocols, Yulianti and colleagues [18] is seeking to develop integrated care practice guidelines specifically for heart disease in pregnancy to strengthen integration of care and referrals from primary to tertiary care facilities in Semarang, East Java. Further studies at the district levels are needed to facilitate effective implementation of new referral protocols and practice guidelines for concomitant illnesses that are contextually appropriate. Consideration may also need to be given in light of the women whom may be experiencing multiple concomitant illnesses in parallel [27]. Another suggested solution by participants to strengthen integration of care was increasing utilisation of telemedicine. Indonesia’s Ministry of Health Strategic Plan from 2020–2024 has planned intentions for the expansion of online referral systems (including for private facilities), telemedicine services and integrated digitalisation of medical records as well as increasing use of online medical records [28].

In respect to the health workforce, participants highlighted that the competency of health providers to diagnose and manage concomitant illnesses in pregnancy remains a challenge. Limited or no training was seen as a contributing factor to a lack of skills by some participants in this study, particularly in sub-health centres and rural and remote areas. Our findings are consistent with other literature in Indonesia that has highlighted that that due to a lack of training, healthcare providers felt there was a gap in their clinical skills to diagnose or manage concomitant illnesses in pregnancy, such as HIV, syphilis and anemia [29, 30]. More broadly, the provision of training for the healthcare workforce remains a challenge across Indonesia. A 2018 World Bank Report highlighted that of primary care staff in *Puskesmas* across Indonesia, a large proportion of staff had not received training for concomitant illnesses and training coverage of staff varied widely by disease, such as STIs (35%), HIV (50%), malaria (55%), cardiovascular diseases (64%), TB (65%), diabetes (65%) [31]. To address this, participants emphasised the need for upskilling and refresher training to be expanded for health providers at all levels of the health system for concomitant illnesses, including obstetricians and specialists, and primary healthcare providers, including midwives, general physicians and community health workers. This could be delivered by identified effective strategies such as tailored interventions to address skills gaps, educational meetings and outreach, practice facilitation, use of local opinion leaders (to influence practices of health providers), audit and feedback for health providers practices and outcomes, and communication training with patients for health providers (specific to the context of the condition) [32]. This will be fundamental for addressing health system challenges regarding integration of care, including but not limited to screening, diagnosis, referral and management for concomitant illnesses in pregnancy. Training and refresher training courses will be useful particularly for primary care staff due to their critical role for the screening and management of concomitant illnesses, and for routine practice of skills. However, training needs to be locally contextual due to variability in disease burdens across Indonesia.

To support integration of care, access to screening and diagnostic infrastructure for concomitant illnesses in primary care needs to be improved, particularly in sub-health centres and rural/remote areas. As evidenced by participants, this plays a pertinent role in identifying cases of concomitant illnesses in pregnancy and referring them to relevant healthcare providers or facilities. Evidence shows that the diagnostic capacity of primary healthcare is still sub-optimal in Indonesia, particularly in rural areas as well as private clinics, with diagnostic tools unavailable for a number of common infectious diseases and NCDs [31]. This should be a priority given evidence has already shown the important role primary care plays for the detection of and management infectious diseases, such as HIV and malaria, for pregnant women in LMICs [30, 33, 34]. A suggested strategy by Beeson and colleagues [27] to deal with concomitant illnesses in pregnancy has been for LMICs to invest in simple, affordable diagnostics in front-line clinics that are able to capture and detect a broader range of diseases. Delivery of rapid diagnostic screening tests by trained midwives situated within integrated health posts (*posyandu)* has been effective by this means in Indonesia [30, 34].

From the perspective of health financing, participants highlighted it is currently a supporting factor of the health system by removing the barrier of cost to access healthcare for pregnant women with and without concomitant illnesses. However, the estimated rise in NCDs will put a significant cost burden on UHC in Indonesia and drive the rise out-of-pocket healthcare expense payments [12]. This will place a strain on the most vulnerable, such as pregnant women and families of low socioeconomic status. With increased demand for care on the health system, funding allocations for concomitant illnesses in pregnancy may be less of a priority.

This study has some limitations. Firstly, we acknowledge that the findings of this study are localised to the perspectives and experiences of key stakeholders in district of Kutai Kartanegara, East Kalimantan. Findings may not be generalizable to other districts given the diverse and varying disease burden and disparities in health system performance across Indonesia. However, these findings may still apply to similar situations across other regions in Indonesia. Secondly, this was a cross-cultural study conducted by videoconferencing software. Some aspects of data may have been lost due to technological disruptions. Some data may also have been missed during translation. Thirdly, we acknowledge that this health systems analysis did not include some stakeholders as initially intended, including women and their families, due to the barriers of the COVID-19 pandemic. Perspectives of health system-end users is pertinent and beneficial for implementation of policy and practice changes to strengthen health systems [35]. However, this presents a future research opportunity.

## Conclusions

This study has identified the supporting factors and challenges of the health system in Kutai Kartanegara district to provide care for concomitant illnesses in pregnancy. In addition, it has highlighted range of suggested improvements for the health system to provide effective, quality care for pregnant women in the face of the growing “double” burden of disease. Beyond this study, further evidence-based research is needed to guide the implementation of policy and practice strategies for the health system to address the growing burden of concomitant illnesses in pregnancy in Indonesia.

## Supporting information

S1 File Indonesian healthcare system. (DOCX)

S2 File Outline of interview guide. (DOCX)

S3 File COREQ checklist and author positionality statement. (DOC)

S4 File Inclusivity in global health research statement. (DOCX)

## Abbreviations

ANC: Antenatal Care
BPJS: *Badan Peyelenggara Jamimnan Sosial*
HIV/AIDs: Human Immunodeficiency Virus/Acquired Immunodeficiency Syndrome
JKN: *Jamiman Kesehatan Nasional*
LMICs: Low and Middle Income Countries
NCDs: Noncommunicable diseases
STIs: Sexually Transmitted Infections
TB: Tuberculosis
UHC: Universal Health Coverage
WHO: World Health Organization
