# Supplementary material for: Concomitant illnesses in pregnancy in Indonesia: A health systems analysis at a District level
Source: PLoS One. 2022 Dec 30;17(12):e0279592. doi: 10.1371/journal.pone.0279592 (PMC9803104; doi:10.1371/journal.pone.0279592)
Supplement: S1 File — (DOCX) [file pone.0279592.s001.docx]

#### Figure 1 Indonesia’s healthcare system


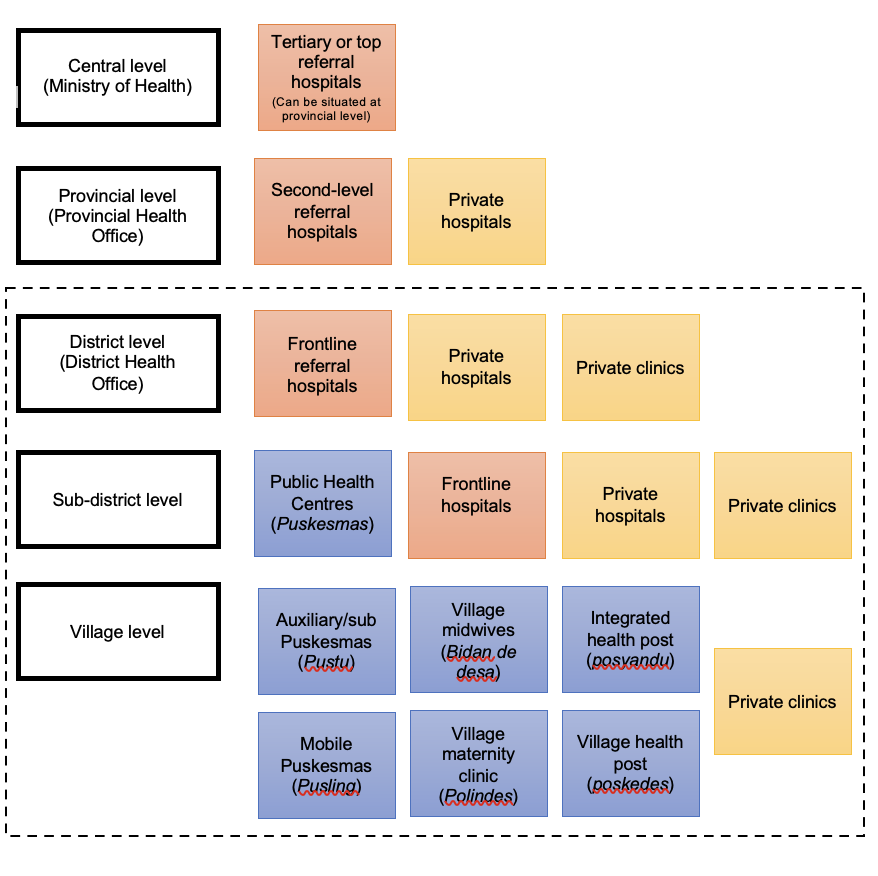


Adapted from Heywood and Harahap; Joint Committee on Reducing Maternal and Neonatal Mortality in Indonesia; Development, Security, and Cooperation; Policy and Global Affairs; National Research Council; Indonesian Academy of Sciences; Mahendradahata and colleagues. [1-3]

**Legend:**

Responsibilities of District Health Office ----

Primary health care services (Public)

Secondary and tertiary hospitals (Public)

Private hospitals and clinics

**Definitions of village level primary healthcare services (from Mahendradahata and colleagues)[3]:**

Auxiliary/sub-puskesmas = simple health service unit; Mobile puskesmas = motorcycle, cars or boats used for remote areas; Village maternity clinic = maternity care (including prenatal care, postnatal care, and family planning); Village health post = Community-based healthcare unit, including village midwives and community health workers (kaders), providing health services including surveillance and health promotion; Integrated health post = Community based healthcare unit providing services for family planning, NCDs, infectious diseases, maternal and child health, and immunization delivered by local kaders, overseen with routine visits from Puskesmas staff.

**Definitions of Primary Health Centres (from Heywood and Harahap; Joint Committee on Reducing Maternal and Neonatal Mortality in Indonesia, Development Security and Cooperation, Policy and Global Affairs, National Research Council, Indonesian Academy of Sciences; Mahendradahata and colleagues)** [**1-3]:** Primary care services provided include curative, rehabilitation, prevention and promotion services. Some health centres may have inpatient facilities for maternity services, open 24 hours providing simple surgery and access to some specialists. Others are considered outpatient facilities, open only during office hours. Other relevant maternal primary health services provided include promotion and prevention, ANC, immunization, family planning, simple laboratory facilities, referrals to specialists, secondary or tertiary care.

**Responsibilities of tiers of government health departments (from Baker and colleagues; Mahendradahata and colleagues [3, 4])**

At a District level, district health departments have autonomy in the governance and operation of health services, healthcare financing, and adaptation of standard operating procedures and guidelines [3, 4]. Provincially, health departments oversee coordination and management of provincial level health services and health budget, while the Ministry of Health centrally oversees for funding of specific health programmes, technical support and guidelines to provincial and district level, and administration of social insurance programmes [3, 4].

**References:**

1. Heywood PF, Harahap NP. Human resources for health at the district level in Indonesia: the smoke and mirrors of decentralization. Human Resources for Health. 2009;7(1):6. doi: 10.1186/1478-4491-7-6.

2. Joint Committee on Reducing Maternal and Neonatal Mortality in Indonesia, Development Security and Cooperation, Policy and Global Affairs, National Research Council, Indonesian Academy of Sciences. Reducing Maternal and Neonatal Mortality in Indonesia: Saving Lives, Saving the Future. Washington DC: National Academies Press (US); 2013. Available from: <https://www.ncbi.nlm.nih.gov/books/NBK201706/>

3. Mahendradhata Y, Trisnantoro L, Listyadewi S, Soewondo P, Marthias T, Harimurti P, et al. The Republic of Indonesia Health System Review. Health Systems in Transition, Vol-7 No.1 ed. New Delhi: WHO Regional Office for South-East Asia; 2017 2017.

4. Baker C, Limato R, Tumbelaka P, Rewari BB, Nasir S, Ahmed R, et al. Antenatal testing for anaemia, HIV and syphilis in Indonesia – a health systems analysis of low coverage. BMC Pregnancy and Childbirth. 2020;20(1):326. doi: 10.1186/s12884-020-02993-x.
