# Supplementary material for: Concomitant illnesses in pregnancy in Indonesia: A health systems analysis at a District level
Source: PLoS One. 2022 Dec 30;17(12):e0279592. doi: 10.1371/journal.pone.0279592 (PMC9803104; doi:10.1371/journal.pone.0279592)
Supplement: S2 File — (DOCX) [file pone.0279592.s002.docx]

**S1 Overview of interview guide**

**Introduction to purpose of project**

Concomitant illnesses in pregnancy, which are pre-existing illnesses or non-obstetric illnesses, are one of the leading contributors to maternal deaths in Indonesia and lower and middle income countries globally. Concomitant illnesses include a broad range of infectious illnesses such as tuberculosis and malaria, as well as non-infectious illnesses, such as heart disease and diabetes.

In Indonesia, this is a particular concern where reproductive-aged women are vulnerable to infectious diseases as well as increasing rates of non-communicable diseases. These diseases are complex to manage, particularly during pregnancy, and require strong integration between multiple health providers and health services.

In the PhD project, analysis is being conducted to understand the burden of concomitant illnesses in pregnancy in Indonesia, and how maternal care may be improved. Therefore, this study aims to explore, from the perspectives of health providers, the facilitators and barriers of the health system to providing care to pregnant women with concomitant illnesses in Indonesia.

Tuberculosis (TB) and pre-existing Diabetes Mellitus (DM) will be used as guiding examples for discussion in this interview, with TB as an existing chronic infectious disease with a high prevalence of TB in Indonesia and DM (pre-existing) as an increasingly common chronic non-communicable disease experienced by reproductive-aged women in Indonesia.

**Question guide**

| **Question** | **Prompt** |
| --- | --- |
| Can you please describe your work and experience in maternal health care in the past and now? |  |
| Can you please describe how care is provided for [tuberculosis/diabetes mellitus] during pregnancy by the health system in your District? |  |
| What health system factors support/help providing care for pregnant women with [tuberculosis/diabetes mellitus]?” | - Protocols/practice guidelines - Coordination and continuum of care - Training - Access to human resource - Transportation - Information systems - Physical resources - Funding |
| What health system factors make it hard/difficult to provide care for pregnant women with [tuberculosis/diabetes mellitus]? | See above. |
| Do you have any recommendations for improving care for [tuberculosis/diabetes mellitus]? | See above. |
| Do you have any recommendations for other concomitant illnesses that may be a concern for pregnant women? | See above. |
| In your opinion, what needs to be improved so that the health system is prepared to provide quality care to pregnant women with concomitant illnesses (whether pre-existing or unknown/identified during pregnancy)? | See above. |
