# Supplementary material for: Concomitant illnesses in pregnancy in Indonesia: A health systems analysis at a District level
Source: PLoS One. 2022 Dec 30;17(12):e0279592. doi: 10.1371/journal.pone.0279592 (PMC9803104; doi:10.1371/journal.pone.0279592)
Supplement: S3 File — (DOC) [file pone.0279592.s003.doc]

**S3 COREQ Checklist and Author Positionality Statement**

**Consolidated criteria for reporting qualitative studies (COREQ): 32-item checklist**

Developed from:

Tong A, Sainsbury P, Craig J. Consolidated criteria for reporting qualitative research (COREQ): a 32-item checklist for interviews and focus groups. *International Journal for Quality in Health Care*. 2007. Volume 19, Number 6: pp. 349 – 357

**Manuscript:** Concomitant illnesses in pregnancy in Indonesia: A health systems analysis at a District level

| **No Item** | **Guide questions/description** | **Section reported in manuscript or description:** |
| --- | --- | --- |
| **Domain 1: Research team and reﬂexivity** | | |
| Personal Characteristics | | |
| 1. Interviewer/facilitator | Which author/s conducted the interview or focus group? | Reported in Methods section of main body text. |
| 2. Credentials | What were the researcher’s credentials? E.g. PhD, MD | See author affiliations. |
| 3. Occupation | What was their occupation at the time of the study? | LR-PhD Candidate, MAM-Academic, IM-Secretary, Department of Health, MY- Director, Department of Health, CL-Academic. See author affiliations for more details. |
| 4. Gender | Was the researcher male or female? | See author affiliations. |
| 5. Experience and training | What experience or training did the researcher have? | LR, the author primarily responsible for conducting interview and data analysis has experience and training in qualitative research methodologies and has experience conducting interviews in cross-cultural settings. She has also undertaken a qualitative methods course and undertaken qualitative research through her Honours degree. |
| Relationship with participants | | |
| 6. Relationship established | Was a relationship established prior to study commencement? | Not applicable. |
| 7. Participant knowledge of the interviewer | What did the participants know about the researcher? e.g. personal goals, reasons for doing the research | Reported in interview guide provided in the Supplementary File. |
| 8. Interviewer characteristics | What characteristics were reported about the interviewer/facilitator? e.g. Bias, assumptions, reasons and interests in the research topic | Reported in interview guide provided in the Supplementary File. |

| **Domain 2: study design** | | |
| --- | --- | --- |
| Theoretical framework | | |
| 9. Methodological orientation and Theory | What methodological orientation was stated to underpin the study? e.g. grounded theory, discourse analysis, ethnography, phenomenology, content analysis | Reported in Methods section of main body text. |
| Participant selection | | |
| 10. Sampling | How were participants selected? e.g. purposive, convenience, consecutive, snowball | Reported in Methods section of the main body text. |
| 11. Method of approach | How were participants approached? e.g. face-to-face, telephone, mail, email | Method of recruitment is described in the Methods section of the main body text. |
| 12. Sample size | How many participants were in the study? | Number of participants is described in the Methods and Results section of the main body text. |
| 13. Non-participation | How many people refused to participate or dropped out? Reasons? | Not applicable. |
| Setting | | |
| 14. Setting of data collection | Where was the data collected? e.g. home, clinic, workplace | Reported in Methods section of main body text. |
| 15. Presence of non-participants | Was anyone else present besides the participants and researchers? | Reported in Methods section of the main body text. |
| 16. Description of sample | What are the important characteristics of the sample? e.g. demographic data, date | Reported in Table 1. |
| Data collection | | |
| 17. Interview guide | Were questions, prompts, guides provided by the authors? Was it pilot tested? | Reported in Methods section of the main body text. |
| 18. Repeat interviews | Were repeat interviews carried out? If yes, how many? | Not applicable. |
| 19. Audio/visual recording | Did the research use audio or visual recording to collect the data? | Reported in Methods section. |
| 20. Field notes | Were ﬁeld notes made during and/or after the interview or focus group? | Not applicable. |
| 21. Duration | What was the duration of the interviews or focus group? | Duration of interviews are described in the Methods section of the main body text. |
| 22. Data saturation | Was data saturation discussed? | Not applicable. |
| 23. Transcripts returned | Were transcripts returned to participants for comment and/or correction? | Not applicable. |
| **Domain 3: analysis and ﬁndings** | | |
| Data analysis | | |
| 24. Number of data coders | How many data coders coded the data? | Reported in Methods section of main body text. |
| 25. Description of the coding tree | Did authors provide a description of the coding tree? | Reported in Table 2. |
| 26. Derivation of themes | Were themes identiﬁed in advance or derived from the data? | Reported in Methods section of the main body text. |
| 27. Software | What software, if applicable, was used to manage the data? | Reported in Methods section of main body text, |
| 28. Participant checking | Did participants provide feedback on the ﬁndings? | Not applicable. |
| Reporting | | |
| 29. Quotations presented | Were participant quotations presented to illustrate the themes/ﬁndings? Was each quotation identiﬁed? e.g. participant number | Reported in Methods section of the main body text. |
| 30. Data and ﬁndings consistent | Was there consistency between the data presented and the ﬁndings? | Reported in results section of main body text. |
| 31. Clarity of major themes | Were major themes clearly presented in the ﬁndings? | Major themes are presented in the Results section and Table 2. |
| 32. Clarity of minor themes | Is there a description of diverse cases or discussion of minor themes? | Reported in Results section of the main body text and Table 2. |

**Author Positionality Statement**

LR, the first author of this paper, primarily responsible for data collection and data analysis of this study identifies an Australian PhD candidate with training in health services research. She has a standing relationship with research collaborators and the local community from 2018. She has prior experience of conducting field research in the district and an understanding of local health systems issues, including related to maternal health. All other co-authors are experienced in the fields of health services management/health systems research (including with a focus on maternal health) within the context of the district, more broadly in Indonesia and/or other developing countries.
